# Matrix metalloproteinase expression in basal cell carcinoma, squamous carcinoma, and melanoma: diagnostic and prognostic potential

**DOI:** 10.3389/fonc.2026.1804092

**Published:** 2026-05-04

**Authors:** Giovanni Francesco Spatola, Alessandro Pitruzzella, Antonino Gioacchino Listro, Carla Valenti, Mariaelena Scalisi, Federica Milia, Paolo Listro, Flavia Zingales, Giulio Maria Ferranti, Marta Listro, Beatrice Belmonte, Domiziana Picone, Giorgia Intili, Maria Laura Uzzo

**Affiliations:** 1 Department of Biomedicine, Neuroscience and Advanced Diagnostics (Bi.N.D), University of Palermo, Palermo, Italy; 2 Centro Dermatologico listro Via Mammana, Palermo, Italy; 3 Laboratory of Pathological Anatomy, Istologi - Via della Caserna 13, Terni (TR), Palermo, Italy; 4 Legal Medicine Unit, University Hospital “Paolo Giaccone”, Palermo, Italy; 5 Istituto di Ricovero e Cura a Carattere Scientifico (IRCCS) Associazione Oasi Maria Santissima (SS) Onlus, Troina, EN, Italy

**Keywords:** basal cell carcinoma, biomarkers, matrix metalloproteinases, melanoma, skin cancer, squamous cell carcinoma

## Abstract

**Introduction:**

Skin cancer, including basal cell carcinoma, squamous cell carcinoma, and melanoma, is one of the most common cancers worldwide. Matrix metalloproteinases are zinc-dependent proteolytic enzymes that play a crucial role in tumor invasion and angiogenesis by degrading the extracellular matrix. This study aims to analyze the specific expression of three matrix metalloproteinases (MMP-3, MMP-9, and MMP-14) to better understand the molecular mechanisms underlying the invasiveness of different skin cancer subtypes.

**Methods:**

The study involved 30 patients aged between 40 and 50 years: 10 diagnosed with BCC, 10 with SCC, and 10 with melanoma. A control group of 10 normal skin samples was also used. All tumor samples were selected from non-invasive, well-differentiated forms. The expression of MMP-3, MMP-9, and MMP-14 was detected using immunohistochemistry on deparaffinized tissue sections.

**Results:**

The IHC analysis revealed distinct expression patterns across the subtypes:

- Basal cell carcinoma: MMP-3 and MMP-14 were strongly expressed with both nuclear and cytoplasmic localization. MMP-9 expression was notably lower and limited to specific cells in the basal layers
- Squamous cell carcinoma: Only weak and diffuse cytoplasmic expression of MMP-14 was identified in the epidermal layers. MMP-3 and MMP-9 were completely absent.
- Melanoma: All three MMPs were highly expressed, exhibiting intense cytoplasmic and nuclear localization. MMP-3 and MMP-14 showed increased expression alongside significantly altered cellular architecture.

**Discussion:**

The differential findings suggest that MMP expression reflects the biological behavior and metastatic potential of each tumor. In Basal cell carcinoma, the significant expression of MMP-3 and MMP-14 suggests involvement in local proliferation and stromal remodeling rather than high metastatic capacity. The absence of MMPs in Squamous cell carcinoma may be due to intense keratinization and a loss of metabolic function in these tumor cells. In melanoma, the high expression of all three enzymes confirms their significant role in aggressive invasion and angiogenesis.

**Conclusion:**

MMP-3 and MMP-14 may serve as valuable diagnostic and prognostic biomarkers, as well as potential therapeutic targets for skin cancer management.

## 1 Introduction

Skin cancer is one of the most prevalent forms of cancer worldwide. It has steadily increasing incidence and mortality rates, particularly in regions populated by fair-skinned individuals. It is strongly associated with chronic exposure to ultraviolet radiation and is classified as either non-melanoma skin cancer (NMSC) or melanoma depending on its aggressiveness and histological origin (1). NMSC is a common tumour with a low mortality rate that originates from keratinocytes and skin adnexa. Basal cell carcinoma (BCC) and squamous cell carcinoma (SCC) belong to this category (2, 3). BCC originates from stem cells in the hair follicle or interfollicular dermis. It predominantly develops in the head and neck regions, less frequently in the trunk and limbs, and rarely in the palms and soles of the feet (4). BCC is classified clinically and histopathologically as either low-risk or high-risk (5), typically presenting as a shiny, pearly, smooth papule or nodule with dilated, arborising blood vessels, erosions or ulcerations, and raised borders (6). SCC originates from keratinocytes and presents clinically as hard, pink or hyperkeratotic papules or plaques that mainly develop on the head, neck and trunk (7). Melanoma is the most aggressive form of skin cancer and originates from melanocytes. It is classified as either primary or invasive based on dermal invasion. Invasive melanoma is further subclassified by clinical and histopathological features into superficial spreading melanoma (SSM), nodular melanoma (NM), lentigo maligna melanoma (LMM) and acral lentiginous melanoma (ALM) (8).

Metalloproteinases are a family of 24 zinc-dependent proteins. They are secreted in an inactive form and are inhibited by a specific category of proteins known as tissue inhibitors of metalloproteinases (TIMPs) (9). These highly homologous proteins share a common structure comprising a pro-domain, a catalytic domain, a signal region and a haemopexin domain. They are classified into different subtypes, including gelatinases (MMP-9), which degrade collagen types 1 and 4; stromelysins (MMP-3), which degrade collagen type 1; and membrane types (MMP-14), which also degrade collagen type 1 (10). In photocarcinogenesis, the degradation of the extracellular matrix (ECM) is the initial step in tumour cell invasion and occurs alongside angiogenic processes (11). MMP-9 plays a pivotal role in tumourigenesis, cell migration, epithelial-mesenchymal transition, and angiogenesis.

MMP-3 is an interesting MMP. Although it plays a significant role in tumour formation, it is expressed at higher levels in the stroma and tumour cells, particularly in more advanced and invasive stages. Its role in skin tumours is ambiguous, particularly in SCC, where its expression is often absent (12). A study by Pittayapruek et al. demonstrated an association between MMP-9 and BCC. This study revealed high MMP-9 mRNA expression in tumour tissues, linking it to more aggressive phenotypes and advanced clinical stages of BCC. This positive association has also been confirmed in head and neck squamous cell carcinoma (HNSCC), where MMP-9 has been identified as a negative prognostic marker, and in oral squamous cell carcinoma, where higher expression was observed in metastatic areas. However, a comparative study of BCC and SCC showed differential expression of MMP-9 between the two tumours, with SCC generally expressing higher levels, particularly in its more aggressive forms. In contrast, MMP-3 is predominantly expressed in the epithelium and stroma of SCC. In the BCC epithelium, however, expression is lower, except in more aggressive forms (13). MMP-14 plays a crucial role in metastatic invasiveness as it is the only MMP capable of degrading fibrillar collagen. Furthermore, it can activate other MMPs, such as MMP-9, and influence the activity of hypoxia-inducible factors (HIFs), thereby facilitating the response to the hypoxic tumour microenvironment (14).

Given the significant role of MMPs in tumour invasion and metastasis, this study aims to analyse the specific expression of MMP-3, MMP-9 and MMP-14 in three distinct skin cancer subtypes: basal cell carcinoma (BCC), squamous cell carcinoma (SCC), and melanoma. Improving our understanding of MMP expression patterns in the main skin cancer subtypes could help to elucidate the molecular mechanisms underlying invasiveness and facilitate the identification of diagnostic and prognostic biomarkers. This could lead to new possibilities for developing targeted therapies for BCC, SCC and melanoma.

## 2 Materials and methods

### 2.1 Sample recruitment

Studies involving human participants were reviewed and approved by the Ethical Review Board of the University of Palermo (approval number 04/2023). In accordance with national legislation and institutional requirements, written informed consent for participation was not required for this study. Thirty patients aged between 40 and 50 years were recruited from the Listro Dermatological Centre in Palermo. Ten patients were diagnosed with basal cell carcinoma (BCC), ten with squamous cell carcinoma (SCC), and ten with melanoma. All samples were selected from non-invasive, well-differentiated forms, representing the least severe cases. For melanoma, the grading was pT1a; BCC was classified as low risk; and SCC was classified as G1. The lesions were identified using video dermatoscopy with epiluminescence, then excised and sent to the Department of Human Anatomy in Palermo for further analysis. For the control group, normal skin samples (CTRL) were collected from ten patients undergoing surgery for non-neoplastic conditions.

### 2.2 Microscopic evaluation of tissue MMPs detected by immunohistochemistry

The sections, which were derived from ten different patients, were deparaffinised in xylene at 60 °C for 30 minutes. They were then rehydrated through a graded alcohol series at room temperature (23 °C). This included a 10-minute soak in 100% alcohol, followed by 5-minute soaks in 95%, 80% and 50% alcohol. This was then followed by a 5-minute rinse in distilled water. Antigen retrieval was performed by heating the sections in 350 ml of sodium citrate buffer solution (pH 6) at 80 °C for eight minutes. To prevent the sections from detaching, they were immersed in acetone at -20 °C for five minutes. All subsequent steps were performed at room temperature (23 °C) using the Immunoperoxidase Secondary Detection System (Millipore, Cat. No. DAB-500). The sections were washed with PBS (pH 7.4) for five minutes, incubated with 3% hydrogen peroxide to block endogenous peroxidase activity for five minutes, and rinsed again with PBS for five minutes. A protein-blocking reagent was applied for five minutes to obstruct non-specific binding sites. The sections were then incubated overnight at 4 °C with the following primary antibodies: anti-metalloproteinase-3 (mouse anti-MMP-3, stromelysin-1, monoclonal antibody, LOT: PSO1482613, 1:150), anti-MMP-9 (mouse anti-MMP-9, H-129, sc-10737, LOT F1110, 1:200) and anti-MMP-14 (Abcam, ab53712, 1:150). After the incubation with the primary antibody, the sections were rinsed with PBS and incubated with a biotinylated secondary antibody (biotinylated goat anti-mouse IgG, Cat. No. 20775, Millipore) for 30 minutes. Burlington, MA, USA & Canada, cat. NDAB-500) for 10 minutes. After another PBS wash, streptavidin HRP diluted in Tris buffered saline (cat. no. 2774, Millipore, Burlington, MA, USA & Canada) was applied for 10 minutes, followed by a final rinse with PBS. Visualisation was performed using Chromogen Reagent. Nuclear counterstaining was obtained using a haematoxylin solution for 15 minutes.

The slides were mounted using a permanent mounting medium (VectaMount H-5000, Vector Laboratories, Inc., Burlingame, CA, USA) and examined using a Leica DMLB microscope with a Nikon DS-Fi1 digital camera. Evaluations were performed at 400× magnification and repeated for ten high-power fields (HPF).

## 3 Results

### 3.1 IHC analysis

### 3.2 MMP-3 expression

**Figure 1:**
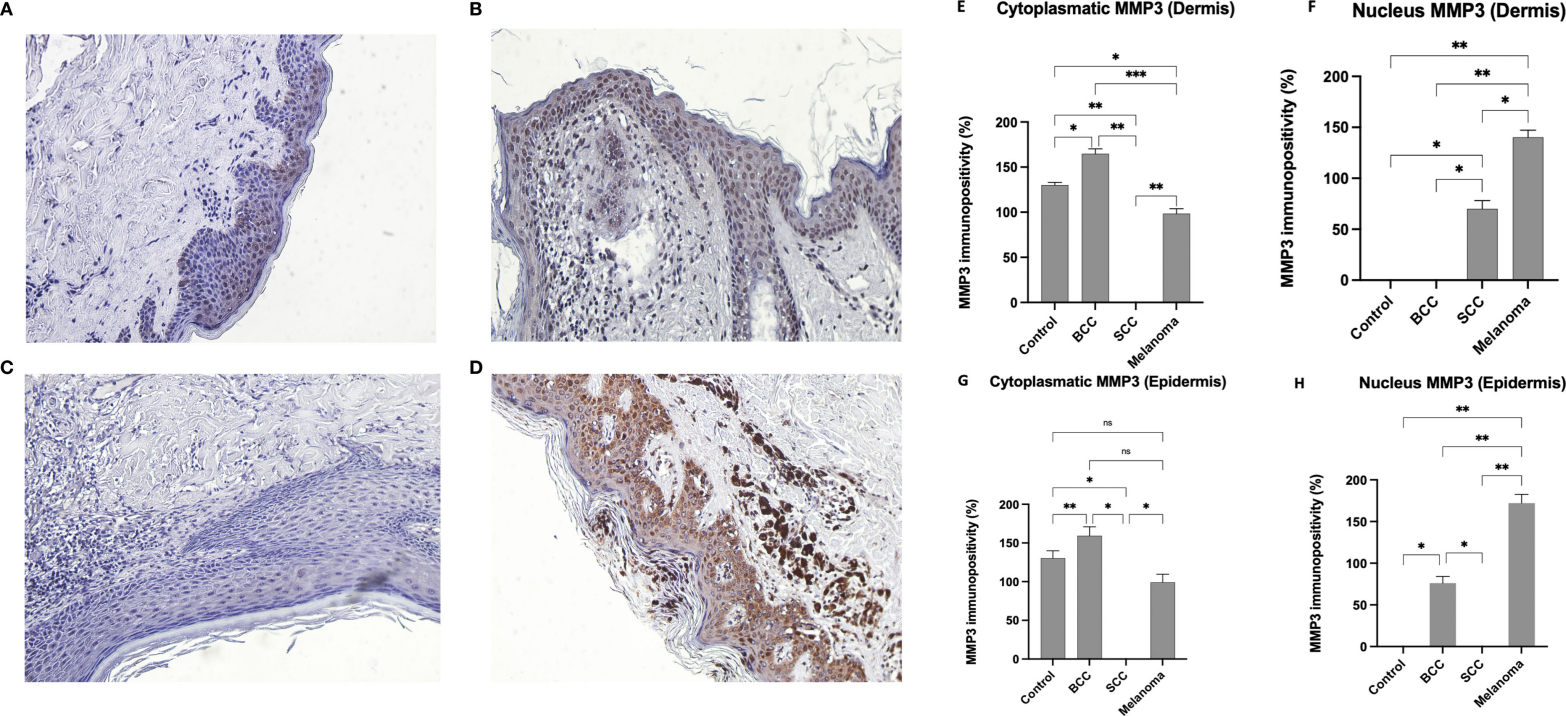
IHC expression of MMP3. Representative images of MMP-3 in CTRL **(A)**, BCC **(B)**, SCC **(C)**, and Melanoma **(D)**. The IHC positivity was analysed using a statistical test known as One-Way ANOVA for multiple comparisons, with Bonferroni’s correction. Magnification was set at 20x. Quantitative Analysis **(E–H)**: Graphs represent the percentage of immunopositivity calculated from mean colorimetric values (where 0 = black and 255 = white). Dermis **(E, F)**: Regarding the dermal compartment, BCC exhibits the highest cytoplasmic expression (approx. 159%), while Melanoma shows a lower cytoplasmic signal (approx. 98%). Conversely, nuclear expression in the dermis is most prominent in Melanoma (approx. 171%), followed by BCC (approx. 76%). In Control and SCC samples, nuclear signal levels are non-detectable (ND). Epidermis **(G)**: In terms of cytoplasmic expression within the epidermis, the highest levels of positivity are found in BCC samples (approx. 193%), followed by Controls (approx. 130%) and Melanoma (approx. 50%). In SCC samples, the cytoplasmic signal in the epidermis is ND. Epidermis **(H)**: Nuclear positivity within the epidermis is highest in Melanoma samples (approx. 147%), followed by BCC (approx. 70%). In both Control and SCC groups, the nuclear signal in the epidermis was found to be non-detectable (ND).

IHC investigation of CTRLs revealed immunopositivity in the superficial layers of the epidermis (see **Figure 1A**), characterised by diffuse cytoplasmic staining and a lack of positivity in dermal cells. Investigations of BCCs revealed a significant number of MMP3-positive cells in various layers of the epidermis and several dermal cells, with exclusive nuclear or perinuclear localisation (see **Figure 1B**). As shown in **Figure 1C**, no MMP3 expression was observed in SCC samples. The pattern of protein expression in melanoma is distinctive and characteristic, with MMP3 appearing to be diffusely present at both the cytoplasmic and nuclear levels (see **Figure 1D**), with increased expression.

### 3.3 MMP-9 expression

**Figure 2:**
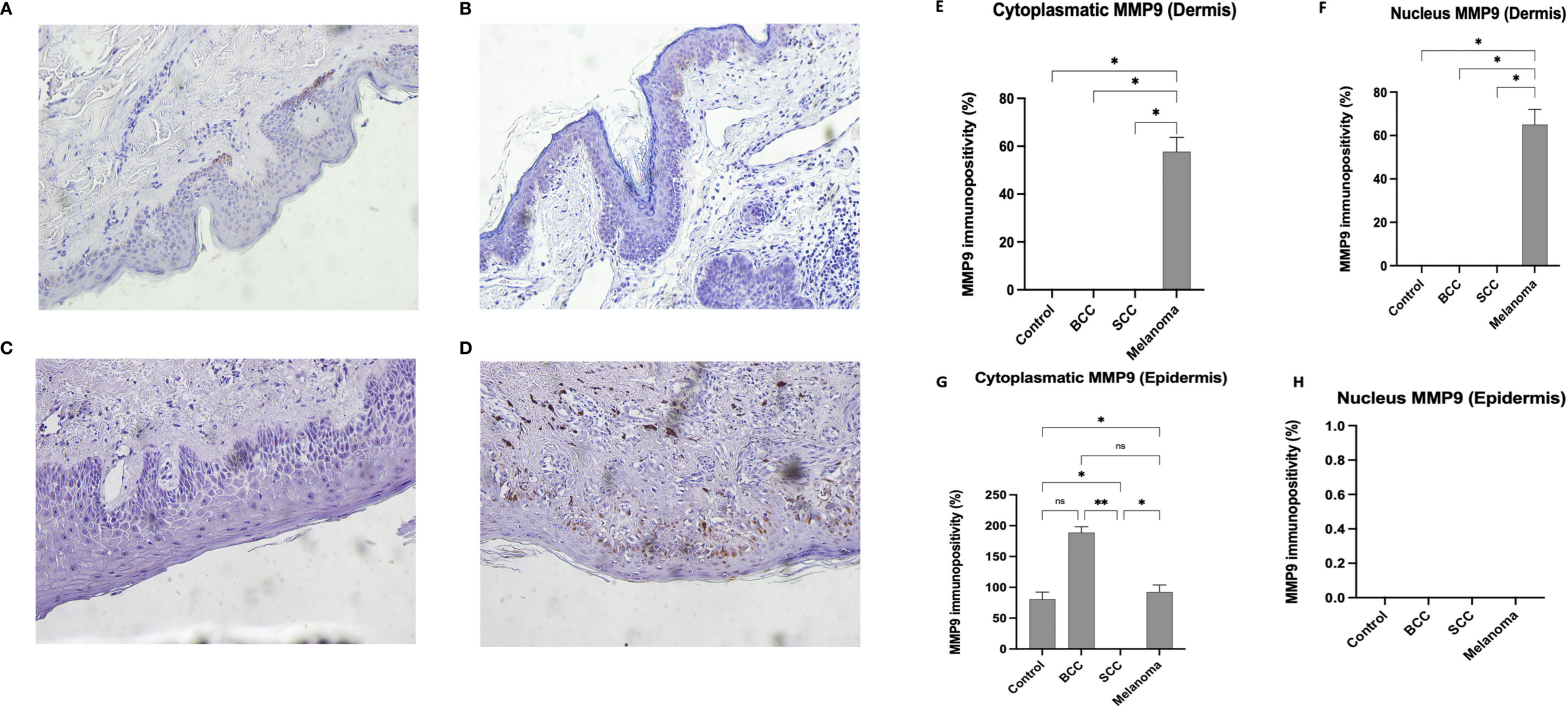
IHC expression of matrix MMP-9. Representative images of MMP-9 in CTRL **(A)**, BCC **(B)**, SCC **(C)**, and Melanoma **(D)**. The IHC positivity was analysed using a statistical test known as One-Way ANOVA for multiple comparisons, with Bonferroni’s correction. Magnification was set at 20x. Quantitative Analysis **(E–H)**: Graphs represent the percentage of immunopositivity calculated from mean colorimetric values Dermis **(E, F)**: MMP-9 expression, both cytoplasmic and nuclear, is exclusive to Melanoma (approx. 57% and 65%, respectively). In Contrast, signal levels in Control, BCC, and SCC samples are non-detectable (ND). Epidermis **(G)**: Regarding cytoplasmic expression, the highest levels of positivity are found in BCC samples (approx. 189%), followed by Melanoma (approx. 92%) and Controls (approx. 82%). In SCC samples, the cytoplasmic signal in the epidermis is ND. Epidermis **(H)**: Nuclear positivity within the epidermis was found to be non-detectable (ND) across all experimental groups.

An IHC investigation of MMP-9 in CTRLs (see **Figure 2A**) revealed cytoplasmic positivity in some cells within the basal layers of the epidermis; no positivity was observed in the dermis. In BCC samples, MMP-9 was expressed only in certain cells in the basal layers of the epidermis (see **Figure 2B**). These elements exhibited weak cytoplasmic reactivity, in contrast to dermal cells, which exhibited no reactivity. **Figure 2C** shows that no MMP-9 expression was observed in SCC samples, in either the dermis or the epidermis. **Figure 2D** shows that MMP-9 expression in melanoma samples appears to be localised to the cytoplasm of cells in the basal layers of the epidermis.

### 3.4 MMP-14 expression

**Figure 3:**
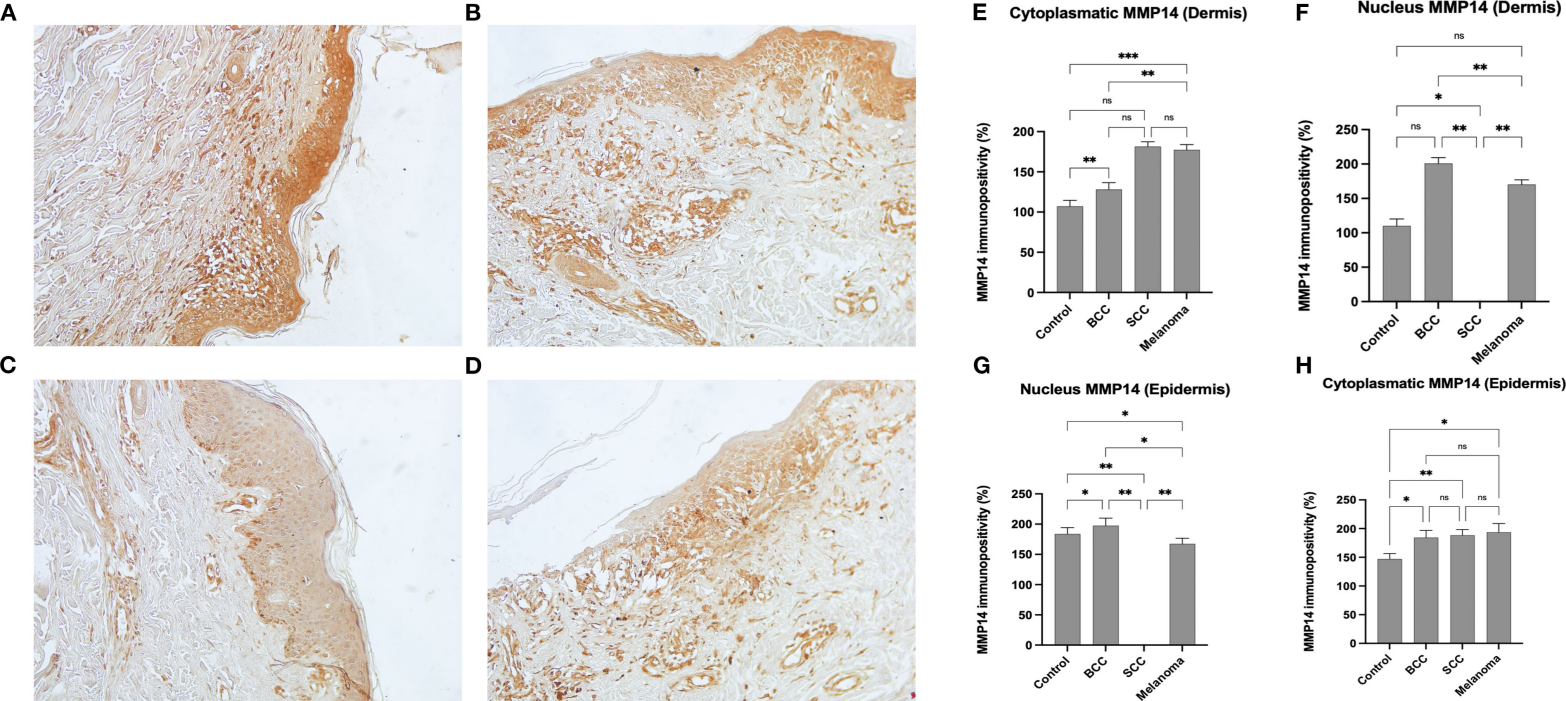
IHC expression of MMP-14. Representative images of MMP-14 in CTRL **(A)**, BCC **(B)**, SCC, **(C)**, and Melanoma **(D)**. The IHC positivity was analysed using a statistical test known as One-Way ANOVA for multiple comparisons, with Bonferroni’s correction. Magnification was set at 20x. Quantitative Analysis **(E–H)**: Graphs represent the percentage of immunopositivity calculated from mean colorimetric values (where 0 = black and 255 = white). Dermis (E, F): Regarding the dermal compartment, the highest cytoplasmic expression is found in SCC (approx. 182%) and Melanoma (approx. 177%), while lower levels are observed in BCC (approx. 127%) and Controls (approx. 106%). In terms of nuclear expression in the dermis, the highest positivity is recorded in BCC (approx. 201%), followed by Melanoma (approx. 177%) and Controls (approx. 110%). In SCC samples, the nuclear signal in the dermis is non-detectable (ND). Epidermis **(G)**: Nuclear positivity within the epidermis reaches its peak in BCC samples (approx. 195%), followed by Controls (approx. 182%) and Melanoma (approx. 167%). In SCC samples, the nuclear signal in the epidermis is non-detectable (ND). Epidermis **(H)**: Regarding cytoplasmic expression within the epidermis, relatively high and similar levels of positivity are observed across all neoplastic groups: Melanoma (approx. 191%), SCC (approx. 189%), and BCC (approx. 184%). Control samples show the lowest baseline cytoplasmic expression (approx. 146%).

IHC staining for MMP-14 showed positivity in all layers of the epidermis in CTRL (see **Figure 3A**), with marked cytoplasmic positivity and presence in some cells of the dermis with predominantly nuclear distribution. In BCC (see **Figure 3B**), MMP-14 is diffusely positive in all layers of the epidermis, with predominant cytoplasmic reactivity and nuclear positivity observed in specific cells of the dermis. In SCC samples (**Figure 3C**), weak and diffuse cytoplasmic positivity was observed in the epidermal layers, with some positive elements present in the spinous layer. **Figure 3D** illustrates that, in melanoma cases, MMP-14 has a prevalent cytoplasmic expression in specific glandular adenomas and in some cellular elements within the deep layers of the epidermis. This invariably occurs together with a markedly altered cellular architecture.

## 4 Discussion

MMPs play a crucial role in extracellular matrix degradation, tumour invasion and metastasis. Their expression varies according to tumour type, stage and microenvironmental conditions, reflecting the biological behaviour of different skin cancers. This study examines the differential expression of MMP-3, MMP-9 and MMP-14 in BCC, SCC and melanoma, providing valuable insights into their potential involvement in tumour progression and invasiveness.

Due to the significant role of MMPs in tumour invasion and metastasis, this study aims to analyse the specific expression of MMP-3, MMP-9 and MMP-14 in three distinct skin cancer subtypes.

In healthy skin, MMP3 positivity is widespread in the superficial layers of the epidermis, with cytoplasmic localisation and an absence in the dermis. This is indicative of its role in physiological maintenance and epithelial renewal. MMP-9 was weakly detected in the cytoplasm of some basal cells of the epidermis. This limited expression of MMP-9 suggests a potential role in regulating the basal microenvironment in response to external stimuli or specific physiological conditions.

Marked expression of MMP-3 has been observed in both the epidermis and dermis of BCC, in both the nucleus and perinuclear regions. This suggests its involvement in local tumour proliferation, but not necessarily in metastatic capacity.

MMP-9 is diffusely expressed in some basal cells and absent in the dermis, which aligns with the low invasiveness characteristic of BCC. MMP14 is strongly expressed in both the epidermal cytoplasm and the dermal nucleus, suggesting a potential role in supporting tumour cell growth and survival even in the stromal environment. In SCC, both MMP-3 and MMP-9 are completely absent at epidermal and dermal levels. This may be due to intense keratinisation and loss of metabolic function in tumour cells. MMP14 showed weak positivity in the cytoplasm of the epidermal layers, particularly in the spinous layer. Although limited, the expression of MMP14 suggests its involvement in tissue support without indicating marked invasiveness.

Compared to other tumour subtypes, the tissue architecture of melanoma is significantly altered, and the marked expression of MMP-3 at the cytoplasmic and nuclear levels suggests it may play a role in tumour proliferation at the transcriptional or regulatory level. The expression of MMP-9 in the basal cells of the epidermis is consistent with its involvement in extracellular matrix (ECM) degradation and, consequently, the invasive potential of melanoma. The expression pattern of MMP-14, on the other hand, is unique. MMP-14 is significantly expressed in the cytoplasm of glandular adenomas and in the deep layers of the epidermis. This abnormal distribution suggests an active role in tissue remodelling associated with tumour progression.

In conclusion, this study aimed to highlight the distinct expression patterns of MMP-3, MMP-9 and MMP-14 across the three skin cancer subtypes. In BCC, significant expression of MMP-3 and MMP-14 in the epidermis and dermis suggests their involvement in local tumour proliferation and stromal remodelling. This is consistent with the tumour’s low metastatic potential. The absence of MMP-3 and MMP-9 in SCC, together with low MMP-14 expression, may reflect the tumour’s keratinised nature and reduced ECM degradation activity.

Overall, these findings support the potential utility of MMP-3, MMP-9, and MMP-14 as complementary molecular markers for improving the characterization of skin cancer subtypes, while highlighting the need for further functional and clinical studies to validate their diagnostic and prognostic relevance.

## Data Availability

The original contributions presented in the study are included in the article/supplementary material. Further inquiries can be directed to the corresponding author.
